# Sodium butyrate as an effective feed additive to improve growth performance and gastrointestinal development in broilers

**DOI:** 10.1002/vms3.250

**Published:** 2020-03-02

**Authors:** Rui Xia Lan, Si Qi Li, Zhihui Zhao, Li Long An

**Affiliations:** ^1^ Department of Animal Science College of Agriculture Guangdong Ocean University Zhanjiang Guangdong P.R. China

**Keywords:** broilers, digestive organ, immune organ, sodium butyrate

## Abstract

This study was conducted to evaluate the effects of dietary sodium butyrate (SB) supplementation on growth performance, the development of gastrointestinal tract and immune organs (thymus, spleen and bursa of fabricius), and serum antibody titer after Newcastle disease (ND) vaccination in broilers. The total of 288 1‐day‐old broilers were randomly allocated to four groups with six replications according to initial body weight. Four treatment groups were designed as follows and fed the indicated diets: CON, basal diet; T1, basal diet supplemented with 0.3 g/kg SB; T2, basal diet supplemented with 0.6 g/kg SB; T3, basal diet supplemented with 1.2 g/kg SB. During days 1–21, broilers fed the T2 diet had higher (*p* < .05) average daily gain (ADG) than broilers fed the CON diet. On day 21, dietary SB supplementation showed linear increase (*p* < .05) in relative weight of the duodenum, jejunum, ileum, small intestine (the sum weight of duodenum, jejunum and ileum), pancreas and thymus, and linear increase (*p* < .05) in relative length of the duodenum, jejunum, ileum, small intestine (the sum length of duodenum, jejunum and ileum) and caeca. Meanwhile, dietary SB supplementation showed linear increase in the antibody titer against ND on days 14, 21, 28 and 35. In conclusion, dietary SB supplementation improved the development of gastrointestinal by increasing the relative weight and length, as well as enhanced the immune response of ND vaccine.

## Graphical abstract

Dietary SB improved the growth performance, the development of gastrointestinal by increasing the relative weight and length as well as enhanced the immune response of ND vaccine.

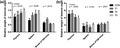

## 1 INTRODUCTION

A common nutritional strategy to increase animal performance is through antibiotics as growth promoters. However, this strategy has been under severe criticism in animal nutrition due to the evolution of resistant strains of bacteria (Witte, 2000). Human health can be directly affected through residues of antibiotic in related food (Boerlin & Reid‐Smith, 2008). The ban on the use of antibiotic growth promoters in feeds and the growing concern of food safety and quality (Kabploy, Bunyapraphatsara, Morales, & Paraksa, 2016; Kleter & Marvin, 2009) encouraged nutritionists to explore antibiotic alternatives to ensure animal performance without compromising human health. For these reasons, the use of organic acids and their salts are generally considered as harmless and beneficial to intestinal health (Moquet et al., 2016; Qaisrani, Krimpen, Kwakkel, Verstegen, & Hendriks, 2015). Butyrate, a short‐chain fatty acid, is a by‐product of microbial fermentation of dietary fibre (Hamer et al., 2008). Butyrate or its sodium salts, as feed additive, is known for its positive effects on growth performance and intestinal integrity (Chamba et al., 2014; Qaisrani et al., 2015; Van Immerseel et al., 2004; Zhang, Jiang, et al., 2011). Meanwhile, butyrate is necessary for the optimum development of intestinal epithelium and gut‐associated lymphoid tissues (Friedman & Bar‐Shira, 2005). Nutritional regulation of sodium butyrate (SB) may bring beneficial effects and provide a simple avenue for promoting gut and immune organs development. Most of the studies on the use of SB were focused on their growth performance, gut morphology, anti‐microbial, immunomodulatory and anti‐oxidative capacities (Liu et al., 2014; Song et al., 2017; Zhang, Gao, et al., 2011; Zhang, Jiang, et al., 2011). However, limited work has been reported the effects of SB on gastrointestinal development and immune organs (thymus, spleen and bursa of fabricius). We hypothesize that dietary SB may improve intestinal health through promoting the development of gastrointestinal tract and the synergetic immune‐enhancing action. Therefore, the objective of this study was to evaluate the effects of dietary SB supplementation on growth performance, the development of gastrointestinal tract and immune organs in broilers.

## 2 MATERIALS AND METHODS

The experimental protocol used in this study was approved by the Animal Care and Use Committee of Guangdong Ocean University (SYXK‐2018‐0147).

### 2.1 Sodium butyrate and vaccine

The sodium butyrate (SB) used in this study was provided by a commercial company (Beijing Shengtaiyuan Biotechnology Co, Ltd.), which contained 54% sodium butyrate and protected by a physical and chemical matrix of buffer salts. ND vaccine (CS_2_ Strain) was purchased from Harbin Pharmaceutical Group Bio‐vaccine Co., Ltd.

### 2.2 Experimental design, animals and housing

A total of 288 one‐day‐old Arbor Acres broilers (144 female and 144 male broilers, respectively) were purchased from a commercial hatchery (Guangxi Liangshan Company) to conduct this 45‐day experiment. All broilers were individually weighed and randomly allocated to four groups (72 broilers per group) with six replications (six female and six male broilers, respectively) according to their initial body weight (BW). Four treatment groups were designed as follows and fed the indicated diets: CON, basal diet; T1, basal diet supplemented with 0.3 g/kg SB; T2, basal diet supplemented with 0.6 g/kg SB; T3, basal diet supplemented with 1.2 g/kg SB. The basal diet was formulated to meet or exceed the nutritional requirements of broilers during starter (days 1–21) and grower (days 22–45) phases according to the NRC (1994) recommendations (Table 1). The SB was supplemented by replacing the equivalent amount of corn meal. All broilers were placed in battery pens (124 cm length × 64 cm width × 40 cm height). The temperature of the room was maintained at 33 ± 1°C for the first week. On day 8, the temperature was gradually reduced by 0.5°C per day until declined to 24°C. Artificial light was provided 24 hr/d by fluorescent lights following a commercial practice and had free access to feed in mash form and tap water through the trial. Broilers were vaccinated using combined Newcastle disease virus (NDV) and infectious bronchitis virus on day 7 via intranasal and intraocular administration and on day 21 through oral administration.

**Table 1:** Ingredient composition and nutrient content of diets (as‐fed basis)

Item	Starter (days 1–21)	Grower (days 22–45)
Ingredients, %		
Corn	54.4	62.3
Soybean meal, 48.0% CP	30.0	25.6
Corn gluten meal, 60.0% CP	5.9	3.3
Soybean oil	5.5	4.9
Tricalcium phosphate	2.5	2.3
Limestone	0.9	0.8
Salt	0.2	0.2
DL‐Met, 88.0%	0.1	0.1
L‐Lys·HCl (78.4%)	0.1	0.1
Vitamin premix#	0.2	0.2
Mineral premix*	0.2	0.2
Calculated composition		
ME, MJ/kg	13.0	12.8
CP, %	21.9	19.0
Ca, %	1.1	1.0
Lys, %	1.1	1.0
Met + Lys, %	0.9	0.9
Available *p*, %	.8	.7
Analysed composition, %		
CP	21.1	20.0
Ca	1.0	1.0
Met + Lys	0.9	0.9
Available *p*	.8	.7

^#^ Provided per kilogram of complete diet: 12,8,000 IU vitamin A, 1,600 IU vitamin D_3_, 60 IU vitamin E, 1.6 mg vitamin K_3_, 0.12 mg biotin, 50 mg choline, 1.2 mg folic acid, 32 mg Nicotinic acid, 16 mg pantothenic acid, 4.8 mg riboflavin, 2.4 mg thiamine (B_1_), 3.2 mg vitamin B_6_ and 0.03 mg vitamin B_12_.

* Provided per kilogram of diet: Mg, 79 mg as manganese oxide; Zn, 60 mg as zinc oxide; Cu, 100 mg as copper sulphate; Fe, 120 mg as iron sulphate; I, 0.96 mg as potassium iodine; Co, 0.16 mg as cobalt sulphate and Se, 0.24 mg as sodium selenite.

### 2.3 Growth performance

BW of broilers and feed consumption were recorded as pen basis on days 1, 21 and 45. Average daily gain (ADG), average daily feed intake (ADFI) and feed conversion ratio (FCR) were calculated by period and cumulatively.

### 2.4 Gastrointestinal tract and immune organs measurements, and digesta pH of intestinal segments

On days 21 and 45, feed was removed 12 hr before sampling, and six female broilers from each treatment (one broiler per pen) were randomly selected for gastrointestinal tract and immune organs measurements. The broilers were individually weighed, killed by cervical dislocation and exsanguinated, then the thymus, liver, spleen, bursa of fabricius, proventriculus, gizzard, duodenum (from the pyloric junction to the distal most point of insertion of the duodenal mesentery), jejunum (from the distal most point of insertion of the duodenal mesentery to the junction with Meckel's diverticulum), ileum (from the junction with Meckel's diverticulum to ileo‐caecal junction) and caeca (from ostium to tip of each) were collected. Prior to digesta emptying, digesta pH was measured within each segment (in the middle part) using a digital pH meter (Model 507, Crison Instruments S.A.). The pH was recorded twice, and the mean was used for statistical analysis. Then the length of each intestinal segment was measured with a flexible tape on a glass surface to prevent inadvertent stretching. The digesta of the proventriculus, gizzard, duodenum, jejunum, ileum and caeca were squeezed, and the empty organs were cleaned with water, dried with filter paper and weighed, and the weight of small intestine is the sum weight of the duodenum, jejunum and ileum. The relative organ weights were expressed as a percentage of live BW (g/kg), and the relative length of the duodenum, jejunum, ileum, small intestine (the sum length of duodenum, jejunum and ileum) and caeca (the sum length of two sides) was expressed relative to live BW (cm/kg) based on former studies by Mahdzvi and Torki (2009) and Ling et al. (2014).

### 2.5 Serum haemagglutination inhibition antibody assay

On days 14, 21, 28 and 35, six female broilers from each treatment (one broiler per pen) were randomly selected for haemagglutination inhibition (HI) antibody titer analyse. Blood samples (2 ml per broilers) were drawn into non‐heparinized vacuum tubes (Becton Dickinson Vacutainer Systems, Franklin Lakes) from the brachial vein and clotted at 4°C for 2 hr. The serum was separated by centrifugation at 3,000*g* for 15 min, and stored at −20°C for HI antibody assay. Briefly, after the serum was inactivated at 56°C for 30 min, twofold serial dilution were made in a 96‐well V‐shaped bottom microtiter plate containing 50 μl of CMF‐PBS in each well, then 50 μl of NDV antigen (4 HA units) was added into all the wells except the last row as the controls. Serum dilutions ranged from 1:2^1^ to 1:2^12^. The plate was incubated at 37°C for 10 min, then 50 μl of 1% rooster erythrocytes suspension was added into each well and continued to incubate for 30 min. A positive serum, a negative serum, erythrocytes and antigens were also included as controls. The last wells which caused complete inhibition was considered as the endpoint. The geometric mean titer was expressed as reciprocal log_2_ values of the last dilution that displayed HI (Thekisoe, Mbati, & Bisschop, 2004).

### 2.6 Statistical analysis

The pen was used as the experimental unit and all data were analysed with SAS 2003 (v. 9.1, SAS Institute Inc.) using the mixed procedure. Orthogonal polynomial contrasts were used to study the linear and quadratic effects of dietary sodium butyrate level. Differences were considered significant at *p < *.05 and tendencies at *p < *.10.

## 3 RESULTS

### 3.1 Growth performance

During days 1–21, broilers fed the T2 diet had higher (*p < *.05) ADG than broilers fed the CON and T3 diets (Table 2). Broilers fed the T3 diet had lower (*p < *.05) ADG and higher (*p* < .05) FCR than broilers fed the CON, T1 and T2 diets. There were no significant differences in ADG, ADFI or FCR during days 22–45 or the overall period of the experiment.

**Table 2:** Effects of sodium butyrate on growth performance in broilers

Item#	Dietary treatments				*SE* *	*p*‐value	
	CON	T1	T2	T3		Linear	Quadratic
Days 1–21							
ADG, g	27.5^b^	27.6^ab^	28.3^a^	25.9^c^	0.25	<.00	<.00
ADFI, g	46.1	46.1	46.7	46.2	0.32	.49	.87
FCR	1.7^a^	1.7^a^	1.7^a^	1.8^b^	0.02	.03	<.00
Days 22–45							
ADG, g	37.2	33.5	35.7	36.3	2.41	.97	.37
ADFI, g	72.3	64.8	71.1	70.0	3.76	.98	.40
FCR	2.0	1.9	2.0	2.0	0.06	.79	.64
Days 1–45							
ADG, g	32.7	30.7	32.2	31.4	1.31	.71	.66
ADFI, g	60.3	56.1	59.7	58.9	2.01	.93	.40
FCR	1.9	1.8	1.9	1.9	0.04	.45	.54

^#^ CON, basal diet; T1, basal diet supplemented with 0.3 g/kg SB; T2, basal diet supplemented with 0.6 g/kg SB; T3, basal diet supplemented with 1.2 g/kg SB; ADG, average daily gain; ADFI, average daily feed intake; FCR, feed conversion ratio.

* SE, standard error.

^a, b, c^ Means in the same row with different superscripts differ (*p < *.05).

### 3.2 Development of the gastrointestinal tract and digesta pH of intestinal segments

Increasing concentration of SB tended to linearly (*p* < .10) increased the relative weight of gizzard and quadratically (*p* < .10) increased the relative weight of caeca on day 21 (Table 3). On day 21, there were linear (*p* < .05) increasing in the relative weight of duodenum, jejunum, ileum, small intestine and pancreas associated with the inclusion of SB. Broilers fed the T2 and T3 diets had higher (*p* < .05) relative weight of duodenum, jejunum, ileum and small intestine than broilers fed the CON diet. Broilers fed the T3 diet had higher (*p* < .05) relative weight of caeca and pancreas than broilers fed the CON diet. On day 45, there was a linear (*p* < .05) increasing in the relative weight of caeca associated with the inclusion of SB. Broilers fed the T2 diet had higher (*p* < .05) relative weight of caeca than broilers fed the CON diet. No significant differences were observed in the relative weight of proventriculus, gizzard, duodenum, jejunum, ileum, small intestine, pancreas or liver.

**Table 3:** Effects of sodium butyrate on the relative weight of digestive organs (g/kg of body weight) in broilers

Item#	Dietary treatments				*SE* *	*p*‐value	
	CON	T1	T2	T3		Linear	Quadratic
Day 21							
Proventriculus	4.4	4.5	4.9	4.9	0.26	.12	.79
Gizzard	14. 6	14.3	16.6	16.6	0.98	.07	.86
Duodenum	3.8^b^	3.8^b^	5.2^a^	5.6^a^	0.32	.00	.62
Jejunum	7.2^b^	8.5^ab^	9.3^a^	8.7^a^	0.43	.01	.06
Ileum	5.4^c^	5.6^b^, ^c^	6.8^ab^	7.7^a^	0.41	.00	.43
Small intestine^b^	16.5^b^	17.8^b^	21.3^a^	22.0^a^	0.94	<.00	.68
Caeca	2.3^b^	2.4^b^	2.2^b^	3.4^a^	0.33	.58	.06
Rectum	0.9	0.9	1.1	0.8	0.10	.97	.10
Pancreas	2.2^b^	2.9^ab^	2.9^ab^	3.7^a^	0.30	.00	.81
Liver	22.5	23.6	22.5	23.2	1.23	.88	.89
Day 45							
Proventriculus	3.8	4.0	3.7	3.5	0.23	.34	.34
Gizzard	18.3	16.8	18.8	17.1	0.81	.68	.90
Duodenum	4.14	4.6	5.6	4.6	0.50	.28	.18
Jejunum	7.7	8.3	9.1	8.1	0.92	.60	.41
Ileum	6.4	6.2	7.7	6.7	0.88	.52	.66
Small intestine	18.2	19.0	22.4	19.5	2.19	.46	.40
Caeca	3.3^b^	4.2^ab^	4.5^a^	4.4^ab^	0.36	.04	.20
Rectum	1.3	1.4	1.4	1.2	0.18	.71	.47
Pancreas	2.6	2.6	2.5	2.7	0.23	.93	.62
Liver	22.5	23.6	22.5	23.2	1.23	.88	.89

^#^ CON, basal diet; T1, basal diet supplemented with 0.3 g/kg SB; T2, basal diet supplemented with 0.6 g/kg SB; T3, basal diet supplemented with 1.2 g/kg SB.

* *SE*, standard error.

^a,b^ Means in the same row with different superscripts differ (*p < *.05).

^c^ Small intestine is the sum of the length of duodenum, jejunum and ileum.

The relative length of duodenum, jejunum, ileum, small intestine and caeca is given in Table 4. On day 21, there were linear (*p* < .05) increasing in the relative length of duodenum, jejunum, ileum, small intestine and caeca associated with the inclusion of SB. Broilers fed the T3 diet had higher (*p* < .05) relative length of duodenum, jejunum, ileum, small intestine and caeca than broilers fed the CON diet. No significant differences were observed in these parameters on day 45.

**Table 4:** Effects of sodium butyrate on the relative length of intestine segments (cm/kg of body weight) in broilers

Item#	Dietary treatments				*SE* *	*p*‐value	
	CON	T1	T2	T3		Linear	Quadratic
Day 21							
Duodenum	34.1^b^	39.4^b^	41.2^b^	55.0^a^	2.99	<.00	.17
Jejunum	74.6^b^	76.0^b^	85.1^ab^	96.1^a^	5.04	<.00	.36
Ileum	72.0^b^	72.2^b^	81.2^ab^	92.8^a^	4.75	<.00	.24
Small intestine^c^	180.6^b^	187.6^b^	207.4^b^	243.9^a^	11.84	<.00	.23
Caeca	34.2^b^	35.8^ab^	38.7^ab^	44.3^a^	3.02	.02	.51
Day 45							
Duodenum	18.3	19.4	18.2	19.0	0.95	.86	.87
Jejunum	40.4	44.6	40.9	41.1	2.68	.91	.46
Ileum	42.6	46.6	46.3	44.4	2.39	.63	.23
Small intestine	101.3	110.6	105.4	104.5	5.48	.85	.36
Caeca	22.2	24.4	24.2	23.8	1.26	.44	.32

^#^ CON, basal diet; T1, basal diet supplemented with 0.3 g/kg SB; T2, basal diet supplemented with 0.6 g/kg SB; T3, basal diet supplemented with 1.2 g/kg SB.

* *SE*, standard error.

^a,b^ Means in the same row with different superscripts differ (*p < *.05).

^c^ Small intestine is the sum of the length of duodenum, jejunum and ileum.

The digesta pH of intestinal segments is given in Table 5. On day 21, pH of duodenum digesta showed linear decreasing trend (*p < *.10) associated with the inclusion of SB, no significant differences were observed in the digesta of jejunum, ileum or caeca. On day 45, pH of jejunum digesta showed linear decreasing trend (*p < *.10) associated with the inclusion of SB, broilers fed the T2 diet had lower (*p* < .05) pH of jejunum digesta than broilers fed the CON diet. No significant differences were observed in the digesta of duodenum, ileum or caeca.

**Table 5:** Effects of sodium butyrate on digesta pH of intestinal segments in broilers

Item#	Dietary treatments				*SE* *	*p*‐value	
	CON	T1	T2	T3		Linear	Quadratic
Day 21							
Duodenum	6.4	6.3	6.2	6.2	0.06	0.05	0.59
Jejunum	6.3	6.3	6.2	6.4	0.08	0.96	0.16
Ileum	6.3	6.3	6.3	6.3	0.14	0.87	0.93
Caeca	6.3	6.3	6.3	6.4	0.13	0.86	0.85
Day 45							
Duodenum	6.2	6.1	6.0	6.1	0.08	0.14	0.50
Jejunum	6.3^a^	6.2^ab^	6.0^b^	6.1^ab^	0.09	0.07	0.21
Ileum	6.1	5.9	6.2	6.2	0.14	0.34	0.54
Caeca	6.1	6.0	6.1	6.1	0.13	0.85	0.86

^#^ CON, basal diet; T1, basal diet supplemented with 0.3 g/kg SB; T2, basal diet supplemented with 0.6 g/kg SB; T3, basal diet supplemented with 1.2 g/kg SB.

* *SE*, standard error.

^a,b^ Means in the same row with different superscripts differ (*p < *.05).

### 3.3 Relative weight of immune organs and ND antibody titer

**Figure 1:**
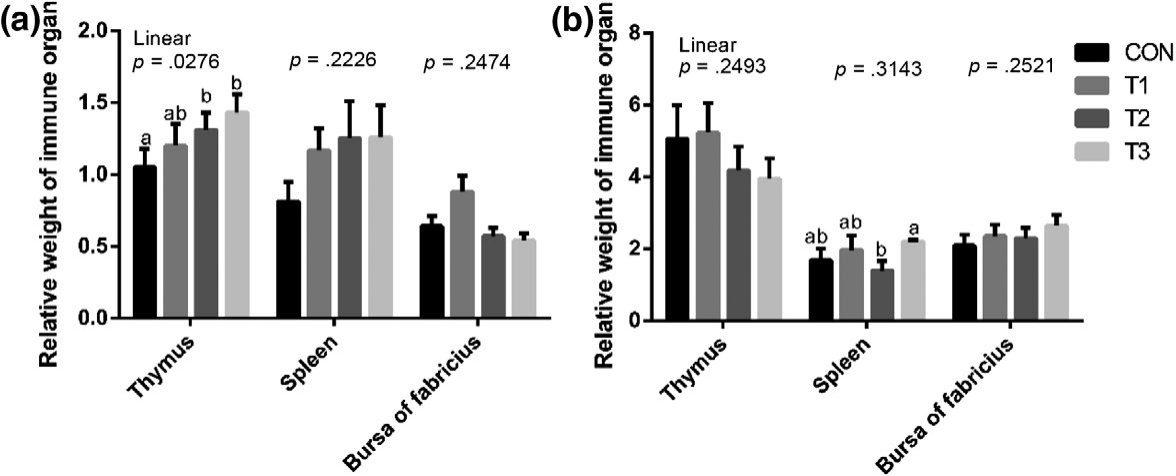
Effects of sodium butyrate on the relative weight of immune organ (g/kg of body weight) in broilers. Values are presented as mean ± *SE*. (a) day 21; (b), day 45; CON, basal diet; T1, basal diet supplemented with 0.3 g/kg sodium butyrate (SB); T2, basal diet supplemented with 0.6 g/kg SB; T1, basal diet supplemented with 1.2 g/kg SB. ^a,b^Means with different superscripts differ (*p < *.05)

The relative weight of immune organs was presented in Figure 1. On day 21, there were linear (*p* < .05) increasing in the relative weight of thymus associated with the inclusion of SB. Broilers fed the T3 diet had higher (*p* < .05) relative weight of thymus than broilers fed the CON diet. No significant differences were observed in the relative weight of spleen and bursa of fabricius. On day 45, broilers fed the T3 diet had higher (*p* < .05) relative weight of spleen than broilers fed the T2 diet. No significant differences were observed in the relative weight of thymus and bursa of fabricius.

**Figure 2:**
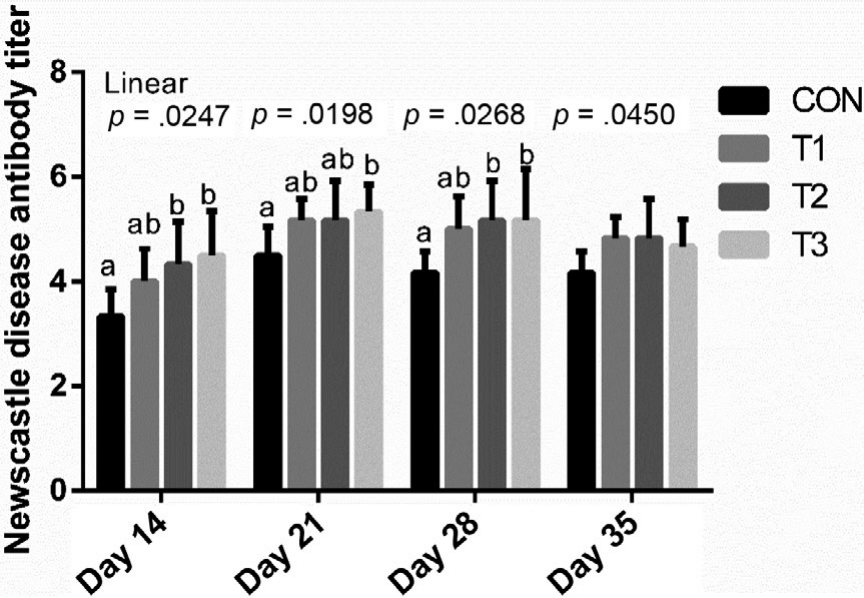
Effects of sodium butyrate on immune Newcastle disease antibody titer in broilers. Values are presented as mean ± *SE*. CON, CON, basal diet; T1, basal diet supplemented with 0.3 g/kg sodium butyrate (SB); T2, basal diet supplemented with 0.6 g/kg SB; T1, basal diet supplemented with 1.2 g/kg SB. ^a,b^Means with different superscripts differ (*p < *.05)

The immune ND antibody titer is presented in Figure 2. On days 14, 21, 28 and 35, there were linear (*p* < .05) increasing in the antibody titer against ND associated with the inclusion of SB. Broilers fed the T2 and T3 diets had higher (*p* < .05) antibody titer against ND than broilers fed the CON diet on days 14 and 28. Broilers fed the T3 diet had higher (*p* < .05) antibody titer against ND than broilers fed the CON diet on day 21.

## 4 DISCUSSION

### 4.1 Growth performance

In this study, dietary 0.6 g/kg SB supplementation had beneficial effects on ADG during days 1–21, which is in consistent with previous reports. Previous reports had indicated that butyrate or its salt form had positive effects on BW gain and FCR (Dehghani‐Tafti & Jahanian, 2016; Sikandar et al., 2017). Conversely, some reports indicated that butyrate or its salt form did not influence the ADG, ADFI or FCR (Leeson, Namkung, Antongiovanni, & Lee, 2005; Mahdzvi & Torki, 2009). There different results may due to the available contents of butyrate, animal age, health status and environment hygiene. Former studies indicated that dietary SB supplementation had no remarkable changes in growth performance of broilers raised in an environment with fewer pathogenic bacteria (Song et al., 2017; Zhang, Jiang, et al., 2011). Additionally, dietary SB supplementation showed decreasing trend of pH in duodenum on day 21 and in jejunum on day 45, the decreasing pH in the small intestine may minimize the load of pathogens and improve digestibility (Hassan, Mohamed, Youssef, & Hassan, 2010), which may explain the better growth performance. Meanwhile, in this study, the SB used was protected by a physical and chemical matrix of buffer salts and can deliver the butyrate in the further distal intestinal tract due to its slow release property, promoting mucosal modulation and stimulating intestinal development (Guilloteau et al., 2010; Wu, Xiao, An, Dong, & Zhang, 2018). The higher relative weight and length of duodenum, jejunum and ileum maybe another reason to explain the better growth performance in the current study.

### 4.2 Development of the gastrointestinal tract and digesta pH of intestinal segments

The relative weight of duodenum, jejunum, ileum, small intestine and pancreas increased as the SB level increased on day 21, which were in agreement with Mahdzvi and Torki (2009), who reported the relative weight of small intestine, jejunum and ileum was increased with SB supplementation. Aghazadeh and Taha (2012) reported higher relative weights of liver and intestine with butyrate, but had no effects on relative weight of gizzard. However, other studies had not indicated effects of butyrate on relative weight of liver or gizzard (Antongiovanni et al., 2007; Panda, Rao, Raju, & Sunder, 2009). In this study, SB tended to linear increase the relative weight of gizzard. A large, well‐developed gizzard improves gut motility (Ferket, Heugten, Kempen, & Angel, 2002) and may increase cholecystokinin release (Svihus & Hetland, 2001), which in turn stimulates the secretion of pancreatic enzymes. A higher pancreas weight was observed with the use of SB as reported in the current study and former studies (Mahdzvi & Torki, 2009; Mallo, Puyalto, & Rao, 2012), the heavier pancreas has also been shown to increase amylase activity in jejunum content, which may improve ileal starch digestibility (Svihus & Hetland, 2001; Svihus, Juvik, Hetland, & Krogdahl, 2004). This increased secretory activity may be due to higher gizzard and pancreas activity. It was suggested that SB stimulates the pancreatic exocrine thus increasing the digestive enzymes, which will improve feed digestion and nutrient absorption, consequently, improving the growth performance.

The relative length of duodenum, jejunum, ileum and caeca was increased as the SB level increased on day 21. Longer jejunum and ileum with SB supplementation has been reported by Chamba et al. (2014). Former studies indicated that butyrate, besides providing epithelial cells with energy, markedly increase the epithelial cell proliferation, differentiation and improve colonic barrier function (Guilloteau et al., 2010). When butyrate was infused in the colon, it exerted trophic effect on ileal and jejunal epithelial cells. In the small intestine, butyrate enhances proliferation, differentiation and maturation, and reduces apoptosis of normal enterocytes through its influence on gene expression and protein synthesis (Sengupta, Muir, & Gibson, 2006). These may be the reasons that the heavier relative weight and length of intestine segments with SB supplementation. Gizzard, pancreas, duodenum, jejunum and ileum are the major organs to produce and release digestive enzymes into the broiler gastrointestinal tract, a higher relative weight of these organs and higher relative length of small duodenum, jejunum and ileum on day 21 may explain the better performance of ADG and FCR during days 1–21.

Increasing concentration of SB tended to linear decreasing (*p < *.1) digesta pH of duodenum on day 21 and jejunum on day 45, respectively. Zou et al. (2010) reported that SB supplementation had no effects on pH value of duodenum, jejunum and ileum. However, limited work has been reported on the effect of SB on pH modulation in the ileum and caeca, which are the major colonization sites of pathogens in poultry. Maintaining low ileum and caeca pH is important for enhancing gut health because gastric acidity can be detrimental to some of the foodborne pathogens residing in the hindgut (Ricke, 2003). Butyrate, a naturally occurring short‐chain fatty acid, is acknowledged as potent inhibiting factors of some pathogenic bacteria. Some studies have demonstrated that the supplementation with butyrate reduced *Salmonella* colonization and shedding, and decreased the occurrence of necrotic lesions in the small intestine induced by *C. perfringens* (Fernández‐Rubio et al., 2009; Timbermont et al., 2010; Van Immerseel et al., 2005). The gastric acidity observed in this study and its effect on intestinal colonization of pathogenic bacteria in broilers needs to be investigated. However, intestinal colonization of pathogenic bacteria was not assessed in this study, which is the limitation of this study.

### 4.3 Relative weight of immune organs and ND antibody titer

In healthy animals, the heavier weight of immune organs is correlated with improved immune response of the body. Spleen is the key player of immune system and the relative weight of spleen in this study linear increased with SB supplementation. In agreement with our study, Sikandar et al. (2017) reported that broilers fed SB supplementation diets had heavier weight of spleen. In poultry, thymus is of vital importance in the differentiation and development of T cells. In young adult mice, about 1% of thymocytes migrate from thymus to periphery per day (Scollay, Butcher, & Weissman, 1980). Moreover, thymus is the place where T cells activities and differentiates to CD4+ or CD8+ T cells, and then mature T cells migrate from thymus to the peripheral blood and secondary immune organs (Erf, Bottje, & Bersi, 1998). Immune organ is the foundation for achieving immune function, and the thymus, spleen and bursa of fabricius are often weighted as parameters to evaluate their critical role in the development and function of the immune system. The immunomodulatory effects of SB in broilers including improving the weight of thymus and spleen (Sikandar et al., 2017). Consistent with former study, in the current study the results show that dietary SB supplementation increased the weight of thymus and spleen.

Serum antibody titer is the indicator of humoral immunity. The changes in antibody titer reflected the state of the humoral immunity in animal organism. This study confirmed that dietary SB could improve the humoral immunity of broilers, thus protecting broilers from attacking of NDV. The level of ND antibody titer is proportional to the livability of broilers challenged by NDV. If the antibody titer is higher, the infection degree of broilers to NDV will be less serious (Ma, Guo, Wang, Hu, & Shen, 2010). Former studies indicated that SB could modulate the function of B and T cells, diminishing the expression of cytokines, such as IL‐6, IL‐10, IFN‐γ and IL‐1β (Ahsan et al., 2016; Zhou, Packialakshmi, Makkar, Dridi, & Rath, 2014)—which might also be one of the immune‐enhancing mechanisms of SB.

In conclusion, dietary SB improved the growth performance during day 1–21, promoted the development of gastrointestinal by increasing the relative weight of duodenum, jejunum, ileum, small intestine, caeca and pancreas, as well as increasing the relative length of duodenum, jejunum, ileum, small intestine and caeca on day 21. Meanwhile, dietary SB enhanced the immune response of ND vaccine in vaccinated broilers.

## CONFLICT OF INTEREST

No potential conflict of interest was reported by the authors.
